# Zoonotic infections in Alaska: disease prevalence, potential impact of climate change and recommended actions for earlier disease detection, research, prevention and control

**DOI:** 10.3402/ijch.v72i0.19562

**Published:** 2013-02-07

**Authors:** Karsten Hueffer, Alan J. Parkinson, Robert Gerlach, James Berner

**Affiliations:** 1 Department of Biology and Wildlife, Institute of Arctic Biology, University of Alaska Fairbanks, Fairbanks, AK, USA; 2 Arctic Investigations Program, National Center for Emerging and Zoonotic Infectious Diseases, Centers for Disease Control and Prevention, Anchorage, AK, USA; 3 Office of the State Veterinarian, Alaska Division of Environmental Health, Anchorage, AK, USA; 4 Community Health Services, Alaska Native Tribal Health Consortium, Anchorage, AK, USA

**Keywords:** Zoonotic infections, climate change, gaps in knowledge, recommendations, US Arctic

## Abstract

Over the last 60 years, Alaska's mean annual temperature has increased by 1.6**°**C, more than twice the rate of the rest of the United States. As a result, climate change impacts are more pronounced here than in other regions of the United States. Warmer temperatures may allow some infected host animals to survive winters in larger numbers, increase their population and expand their range of habitation thus increasing the opportunity for transmission of infection to humans. Subsistence hunting and gathering activities may place rural residents of Alaska at a greater risk of acquiring zoonotic infections than urban residents. Known zoonotic diseases that occur in Alaska include brucellosis, toxoplasmosis, trichinellosis, giardiasis/cryptosporidiosis, echinococcosis, rabies and tularemia. Actions for early disease detection, research and prevention and control include: (1) determining baseline levels of infection and disease in both humans and host animals; (2) conducting more research to understand the ecology of infection in the Arctic environment; (3) improving active and passive surveillance systems for infection and disease in humans and animals; (4) improving outreach, education and communication on climate-sensitive infectious diseases at the community, health and animal care provider levels; and (5) improving coordination between public health and animal health agencies, universities and tribal health organisations.

Over the past 50 years, Alaska's average annual temperature has increased by 1.6**°**C which is more than twice the rate of the rest of the United States. Observed effects include an increase in precipitation, and length of frost-free growing season, a decrease in sea ice, glacial and snow cover, an increase in sea level, an increase in river outflow, changes in plant and vegetation cover with an increase in shrubs and vascular plants and change of tundra and permafrost to a wetlands ecology, and a decrease in permafrost in some regions with consequential damage to built infrastructure (1,2). These changes are of concern to those residents of Alaska who depend on the local environment for water, key subsistence food wildlife species, such as terrestrial and marine mammals (caribou, reindeer, moose, muskoxen seal and walrus), fish, water fowl and plants) for their health and cultural well-being. Climate and weather also affect the distribution, and risk of many helminthic, bacterial, viral and protozoan parasitic diseases as well as insect and ectoparasitic vectors affecting both animals and humans. However, the interaction of hosts, vectors, pathogens and the environment are poorly understood in the Arctic (3). While most pathogens are species-specific, that is, restricted to either certain animals or humans, some, i.e. the zoonoses, are transmissible from animals to humans, often through close contact with animals or food products derived from these animals. Zoonotic infectious diseases may be climate sensitive. Warmer temperatures may allow a parasite to survive in the environment for longer periods, increase an insect replication cycle, allow an infected host animal species to survive winters in larger numbers, increase in population and expand their range of habitation, thus increasing the opportunity for transmission of the infection to humans. Subsistence hunting, gathering, food preparation and preservation methods may also place people at risk for acquiring zoonotic infections. Known zoonotic diseases that occur in Alaska include brucellosis, toxoplasmosis, trichinellosis, giardiasis, cryptosporidiosis echinococcosis, rabies and tularemia. This article reviews what is known about the potentially climate-sensitive zoonotic infectious diseases of concern in Alaska, identifies common gaps in our knowledge of these pathogens and recommends actions that will begin to address earlier disease detection, research and prevention and control of these potential zoonotic disease threats in the context of a changing Alaskan climate.

## Brucellosis

Brucellosis is a systemic disease caused by a number of different species of bacterium (*Brucella*) that may result in abortion, infertility and chronic infections in animals. The bacterium can be passed from domestic and wild animals to humans via ingestion of raw milk products, exposure to fluids and tissues of infected animals, through cuts or abraded skin and exposure by infectious aerosols via lungs and eyes. There are 10 species of *Brucella* recognised in animals and some include different strains or biovars. Three main species are known to cause illness in humans: *Brucella abortus* (from cattle and bison), *B. melitensis* (from sheep and goats) and *B. suis* (from pigs, caribou and reindeer). *B. suis* biovar IV have been found in caribou and reindeer (4). Eating raw, undercooked, dried, smoked or frozen meat or organ tissue (liver, kidney, bone marrow) can expose people to the infection. Brucellosis is a systemic bacterial disease of acute or insidious onset with continued intermittent or irregular fever of variable duration with headache, weakness, sweating, chills, joint pain and weight loss. The first human case of *B. suis* biovar IV was detected in Alaska in 1959 and was linked to the consumption of raw caribou (5). Since then, human infections have been linked to the consumption or processing of raw caribou meat, and infection has been shown to be endemic in many caribou and reindeer herds across Alaska and northern Canada (6–8). This is in contrast to other parts of the United States, where the most common route of transmission is through the consumption of raw dairy products and meat. However, there is little data on the prevalence of brucellosis in humans or wildlife in Alaska. More contemporary data on the prevalence of brucellosis in humans and wildlife in Alaska are needed. Other *Brucella* spp. can also infect other land and marine mammals (9,10). While human infections caused by a marine *Brucella*, found in seals (*B. pinnipedialis*), has also been documented (11), evidence for the direct transmission from seals to humans has yet to be established, as is the risk to humans who depend on marine mammals as a food source. In Alaska, human *Brucella* infections are reportable conditions to the State of Alaska, Division of Public Health, Section of Epidemiology (SOE) and infections in wildlife, domestic herds and other animals are reported to the Office of the State Veterinarian (OSV). Since 1973, there have been 17 reported human cases in Alaska (12,13). Reporting in animals is complicated by the absence of standardised diagnostic tests for different species of animals. Current serologic tests are not species-specific and cross-react with other bacteria and *Brucella* spp underscoring the need for the development of standardised, validated *Brucella* specific tests. Efforts are underway to establish and validate simple filter paper blood collection systems that can be used by hunters and wildlife biologist for wildlife in the field (14) for the detection of *Brucella* antibody using serology. Preventive measures include vaccination of domestic livestock (cattle, reindeer), community education recommending fully cooking meat, cleaning of surfaces and tools with a water/bleach solution after contact with meat. Hunters should take special precautions when butchering animals in the field, such as careful examination of the animal carcass for abnormalities suggesting brucellosis, wearing gloves and glasses to avoid being infected by animal blood through cuts and abrasions on hands or contact with the eyes or mouth.

It is unclear if or how climate change may influence brucellosis rates in animals or people. A warming Arctic may change the range of many animals, contribute to the greater survival of infected animals during winter, which in turn could either increase or decrease herd densities and contribute to greater comingling of species, thus increasing the potential for greater interspecies transmission and transmission to humans. Conversely, changes in weather patterns (more snow and ice cover) may limit access to important food sources, affecting animal nutrition resulting in decreased herd densities, fewer animals to harvest and reduced opportunities for human infection with *Brucella*.

## Toxoplasmosis

Toxoplasmosis is caused by infection with *Toxoplasma gondii*, a widespread protozoan parasite of mammals and birds. Members of the cat family are the only known definitive host of *T. gondii*. However, the asexual encysted stage can be found in muscle tissues of other animals and can serve as the main reservoir of infection in cat-free areas. Humans become infected by ingesting raw or insufficiently cooked meat or foods that have come into contact with viable oocysts from soil, or items that have come into contact with cat faeces (unwashed vegetables). Typically, the infection in humans is mild, with fever and lymphadenitis or can be asymptomatic. In immune-compromised individuals, more severe complications such as encephalitis or acute toxoplasmic chorioretinitis may occur. However, the main health concern is in pregnant women where tachyzoites can migrate transplacentally resulting in foetal morbidity or mortality.

A serosurvey was conducted in the 1960s and 70s which showed that 15% of Alaska Natives had antibodies to *T. gondii*, a rate similar to that found in the United States (15). This contrasts with a more recent study conducted among the Inuit of Nunavik which showed a seroprevalence of 60%, almost three times the North American average prevalence (16). Because felid hosts are rare in this region, it is likely that the source of infection is related to the eating of raw, dried or undercooked meat (caribou, land or waterfowl or seal (17)). The recent discovery of *Toxoplasma* in polar bears and Arctic foxes in Svalbard in the absence of a definitive host also highlights the existence of alternative transmission routes. (18). It has been hypothesised that *Toxoplasma* in animals in this region may have been introduced to this region by migratory birds. The prevalence of *Toxoplasma* in polar bears in Svalbard, the Barents Sea Region and Eastern Greenland areas has doubled in the last decade (now 46%), and detection in Ring Seals for the first time highlights the role of predator–prey cycles as a mechanism of maintaining this infection in this region, and the risk of infection in human populations that rely on marine mammals for food (19).

In the United States, toxoplasmosis in humans is neither a national nor a state-mandated reportable condition; therefore detailed statistics about the disease in Alaska are limited. Seroprevalence studies in humans may provide information about the disease exposure in Alaska.

The disease in wildlife domestic herd and other animals is reportable to the OSV. However, better surveillance data for *Toxoplasma* in wildlife species is needed to understand the ecology of the disease in Alaska. Two diagnostic tests are available for *T. gondii* that can be used in various species of animals – a molecular method (multiplex PCR) and a multi-species enzyme immunoassay (ELISA) that detects its antibodies in blood or tissue fluid. An absorbent filter paper method to collect blood from animals under field conditions for the diagnosis of *T. gondii* in wildlife is being developed (20,21). This will simplify blood collection under Arctic conditions and facilitate sample collection by hunters.

Measures to prevent human infection include: fully cooking meat, washing fruits and vegetables, cleaning food preparation surfaces with a water/bleach solution after contact with meat. Hunters and trappers should take special precautions when hunting or trapping lynx, as lynx serve as the only known Alaska wildlife source of *Toxoplasma* oocysts. There is a need for more public education, particularly among pregnant women in Arctic regions to alert people to the fact that this is a food-borne disease, and is not always associated with cats (22). Since testing began on samples collected from Alaska wildlife in the 1970s, antibodies against *T. gondii* have been detected in a wide variety of species, including lynx, black bears, grizzly bears, wolves and herbivores (23–25). Among marine mammals, antibodies have been detected in walrus, Steller sea lions, harbor, ringed, spotted and bearded seals (26).

While there is no evidence yet of increased transmission of *toxoplasma* associated with climate change effects, there are climate and weather effects that may either increase or decrease the survival of parasites in the environment, increase or decrease the abundance of host species, and may result in the emergence of disease at altered interfaces among wildlife, domestic animals and human populations. For example, higher humidity and warmer soil temperatures may increase the rate of sporulation and survival of oocysts (27). Increased storm events predicted with climate change are likely to result in damage to community water intake systems, increased runoff into rivers or other fresh water sources and increased chances of contamination of community water supplies with oocycts (28). Storm related runoff also increases water turbidity, frequently compromising water purification filter systems. This together with damage to water and sanitation infrastructure caused by melting permafrost potentially increases the chance of water borne outbreaks of toxoplasmosis in these communities. The dramatic loss of Arctic sea ice may result in animal crowding on land and remaining sea ice, increased mortality and scavenging that may increase the transmission of oocysts to carnivores, marine shellfish and thence to other marine mammals, whales, walrus, otters and seals, which are all important substance species for Alaska Natives (29).

## Trichinellosis

Trichinellosis is caused by any species of *Trichinella*, an intestinal roundworm whose larvae migrate and become encapsulated in muscle tissue of domestic (commonly pigs) or wild animals. In the Arctic, bear and walrus meat are the main sources of human infection. The most common species in Arctic regions are *T. nativa* and *Trichinella* genotype T6 which, unlike other *Trichinella* species, survives freezing. Infection in humans occurs following consumption of raw or undercooked meat containing viable encysted larvae. Illness in humans is variable ranging from asymptomatic infection to a rarely fatal disease, depending on the number of larvae ingested.

The most frequent reports of human trichinellosis in Alaska are multi-person outbreaks that result from more than one person consuming the same meal of under-cooked bear, walrus or seal meat (30). In Alaska, from 1974 to 1994, 203 cases of *Trichinella* were reported to the SOE. Of the 34 outbreaks documented, 20 (59%) were caused by the consumption of bear meat, 13 (38%) from walrus meat and in one case (3%) from the consumption of either walrus or seal meat. Most outbreaks of trichinellosis in Canada have been due to *Trichinella nativa* or Trichinella genotype T6 which is generally found in game animals such as black bears, grizzly bears, polar bears and walrus (31,32) In Alaska, *Trichinella* infections are reportable to SOE and the OSV. *Trichinella* can be detected in muscle tissue by laboratory methods that use artificial digestion of muscle tissues followed by microscopy or DNA analysis by PCR. Microscopy introduces the possibility of false negative result if small numbers of larvae are present. Antibodies to *Trichinella* can be detected by ELISA. However, antibody cross-reactivity with antigens of other parasites may give false positive results, underscoring the need for more species-specific antibody assays (33).

Prevention messages should stress that *T. nativa* in bear and walrus meat is cold resistant and that meat should be cooked to a temperature of at least 160**°**F. Smoking, salting, fermenting or drying meat are not reliable methods for killing the parasite (34). Enhanced surveillance for *Trichinella* in subsistence food animal populations in Alaska would help inform prevention efforts. A model programme exists in Nunavik where walrus harvested by hunters is tested before consumption (35). However, this requires a sustainable source of funding, a system for rapid testing and rapid communication of results back to the community.

Climate change may affect the migration of terrestrial or marine mammals. This could result in changes in the availability of food, quality and species composition of food, and increase in contact between terrestrial and marine mammals. Loss of sea ice could interfere with resting, feeding and breeding of marine mammals (29). The decline of summer sea ice has already resulted in large “haul-outs” of walruses on beaches in northwestern Alaska. Dead animals on beaches or on ice packs or at the bottom of the sea and may contribute to the transmission of *Trichinella* to other land or marine scavengers and carnivores (29,36). There is no current evidence that climate change has contributed to an increase in *Trichinella* prevalence in Alaskan wildlife populations.

## Giardiasis and cryptosporidiosis

Giardiasis is caused by infection with *Giardia* protozoa (*Giardia intestinalis*), and in humans it is typically characterised by self-limited diarrhea (37). Cryptosporidiosis, also a self-limited diarrheal disease in humans, is caused mainly by infection with *Cryptosporidium parvum* or *C. hominis*. Fatalities can occur in widespread outbreaks where the elderly or immunosuppressed are infected. Both *Giardia* and *Cryptosporidium* protozoa are widespread in the environment. While *Cryptosporidium* infections are often associated with the consumption of untreated surface water contaminated with animal faeces, *Giardia* is most commonly associated with the ingestion of water contaminated by human faeces. Recently characterised molecular variation among *Giardia* and *Cryptosporidium* parasites has allowed genotypes to be divided into assemblages, which are grouped by degree of similarity (38,39). Data suggest that among *Giardia* protozoa, certain assemblages are more likely to be associated with specific animal species, and parasites found in humans are more likely to align with assemblages consisting of isolates from other humans, as opposed to parasites from non-human animal species. This might mean that person-to-person transmission, an acknowledged but not typically emphasised route, is more common than previously expected, as opposed to transmission being primarily from a zoonotic or environmental source.

In Alaska, human cases of both *Giardia* and *Cryptosporidium* are reportable to the SOE. From 2001–2010, 1,042 human cases of giardiasis were reported, and annual rates of giardiasis in Alaska are routinely higher than in the rest of the United States (37). The number of reported giardiasis cases underestimates the true incidence of disease by an unknown amount, as only laboratory-confirmed infections are typically reported to SOE and empiric treatment of suspected cases based on clinical presentation are not usually reported to SOE.

In contrast, human disease caused by *Cryptosporidium* is rare and ranges from 0 to 4 cases per year in Alaska. However, it is also likely that it is under-diagnosed. The State Public Health Laboratory currently conducts testing of stools. New serologic tests are becoming available that could be used to determine seroprevalence of exposure at the community level.

Wildlife and domestic animal disease caused by *Giardia* or *Cryptosporidium* are reportable to the OSV but is most likely under-reported by practitioners unfamiliar with regulation and animals are often treated clinically rather than diagnoses due to financial limitations. The prevalence of *Cryptosporidium* and *Giardia* spp. appears to be widespread among wildlife in Alaska (39,40). Either boiling or filtering untreated surface water before drinking can prevent giardiasis and cryptosporidiosis. It is important to protect public water supplies against contamination with animal or human faeces and ensure adequate filtration of community water supplies.

Giardiasis and cryptosporidiosis may be sensitive to climate change. Higher temperatures and a changing environment may favour the migration of host animals northward into regions where disease has yet to be established. In Alaska, the tree line has moved northward as have beaver and muskrat populations, potentially exposing human residents of these regions to new diseases such as *Giardia* or other diseases carried by insect vectors associated with these animals (41). Climate change may bring precipitation and increased soil moisture, which may increase pathogen survivability. Increased runoff into drinking and recreational water sources may increase the concentration of organisms and potential for transmission. Increased storm events may result in damage to community water intake systems, increased runoff into rivers or other fresh water sources, increasing the chance of contamination of community water supplies (29). Storm related runoff also increases water turbidly, frequently compromising water purification filter systems. This together with damage to water and sanitation infrastructure caused by melting permafrost potentially increases the chance of water borne outbreaks of giardiasis and cryptosporidiosis in these communities.

## Echinococcosis—cystic and alveolar hydatid disease

*Echinococcosis granulosis* has worldwide distribution (42) and there are two recognised biotypes: northern (or sylvatic) and the domestic biotype. Northern strain cystic hydatid disease is caused by *Echinococcus granulosus* which maintains a cycle that includes an adult cestode stage in the definitive host such as a wolf, coyote, fox or dog, and a larval cestode stage in an intermediate host such as a moose, deer, caribou, reindeer but does not cross-infect domestic livestock (43). Humans usually acquire the infection via exposure to eggs that were shed in canid faeces and are an accidental host (43). The infection caused by the northern biotype in humans is relatively benign (44), developing over many years and resulting in cyst formations in the liver and lungs (42,44). In contrast, human alveolar hydatid disease caused by *Echinococcus multilocularis* is associated with a much higher mortality rate than cystic hydatid disease. Alveolar hydatid disease has a distribution throughout the northern hemisphere (42) and is endemic in south-central Canada and the northern Midwestern United States (45). *Echinococcus multilocularis* maintains a cycle that includes an adult cestode stage in the definitive host, commonly a fox, wolf, coyote or dog, and a larval cestode stage in an intermediate host such as a vole or a deer mouse.

In Alaska, cases of human disease caused by *Echinococcus* species (both *granulosus* and *multilocularis*) are reportable to the SOE. Since the 1950s, over 300 cases of echinococcosis were reported (46). Since 1990, only eight cases of *E. granulosus* and no cases of *E. multilocularis* have been reported (46). Because cases may be asymptomatic, the actual number of infections could be higher. In Alaska, there were 193 (mostly Alaska Native) cases diagnosed up until 1980, and various reports document similar numbers from various regions of northern Canada (47).

In 1990, high human rates of alveolar hydatid disease were reported from western Alaska (especially Saint Lawrence Island). In these villages, the annual incidence of diagnosis ranges from 7 to 98/100,000 (48). Between 1951 and 1991, 70 cases of alveolar hydatid disease were diagnosed mostly from Northwestern Alaska and in Alaska Natives (49).

More specific serologic tests for diagnosis and seroprevalence studies are needed. New molecular typing methods are being developed (50), but currently little is known about the significance of molecular variation. Dog treatment programmes in the 1980s significantly reduced the prevalence in dogs (51). However, many of these programmes have since been discontinued. An assessment of these remaining programmes is needed to improve educational outreach, community based control programmes and guidelines for dealing with dogs. Prevalence of the domestic biotypes in the domestic livestock populations is also unknown. A survey of infection rates in animal reservoirs is needed in order to identify geographical distributions and to lay a baseline for future climate related fluctuations in reservoir populations. This would require local laboratory capacity, mapping resources, trained technical staff and funding.

Prevention measures include: the education of hunters and trappers to use gloves, hand washing and to practice good food hygiene and appropriately dispose of viscera; education of community members on modes of transmission and good personal hygiene; ensure communities practice proper dog control (i.e. keeping dog lots away from the community) and institute dog treatment (Praziquantel) programmes.

*E. granulosis* and *E. multilocularis* are already present in the Arctic and their distribution seems to be dependent on the presence of the appropriate host. A warming Arctic may increase egg survival in the environment, alter distribution and abundance of intermediate hosts and alter interfaces among wildlife, domestic animals and people. (29).

## Rabies

Rabies is an acute viral infection causing progressive viral encephalomyelitis that is nearly always fatal. Rabies is caused by RNA viruses in the family *Rhabdoviridae*, genus *Lyssavirus*. Transmission is most often through saliva via a bite from an infected animal. Mammals are the natural hosts of rabies. Reservoirs consist of the *Carnivora* (canids, skunks, raccoons, mongooses) and *Chiroptera* (bats). Dogs are the main source of infection for humans, although wildlife is also an important source of infection in developed countries where dog rabies has been mostly eliminated. Rabies is enzootic among the fox populations of northern and western Alaska, with periodic epizootics documented every 3–5 years following variation in prey and predator populations (52). The last epizootic in Alaska was in 2006–07. Although rabies has been documented in animals every month of the year, most cases are usually reported during early fall through early spring. In early spring, Arctic foxes tend to move inland off the sea ice, increasing the likelihood of coming into contact with domestic animals or humans. Dogs can readily serve as a transmission vehicle of the rabies virus from wildlife to humans; therefore, it is especially critical during these seasons to ensure that adequate rabies prevention and control measures are in place. The prevalence of rabies in wildlife reservoirs is unknown and reducing rabies in the Alaskan fox populations is not yet feasible. Therefore, the mainstays of preventing human rabies cases are the use of a non-veterinarian lay vaccinator programme in rural areas, public education of the risk in the wildlife (fox) population, vaccination of domestic animals against rabies and prompt administration of rabies post-exposure prophylaxis to persons potentially exposed to the rabies virus (53).

Human cases and potential exposures are reportable to the SOE which are promptly investigated. Three human cases have been reported since 1914, but none since 1942. Between 15 and 50 cases in wildlife are reported each year. Many gaps in our understanding of this disease remain, and areas requiring further investigation include: (1) factors influencing epidemics in animals (epizootics); (2) the lack of evidence of animal rabies in the interior of Alaska; (3) the ecology and interaction between Arctic and Red foxes; and (4) the potential impact of climate change on disease and the role of species incursions (red foxes and bats). While standard fluorescent antibody microscopy diagnostics are available at Alaska State Virology Laboratory in Fairbanks, a key challenge is the lack of the full range of in-state veterinary diagnostics. Some surveillance is currently underway by the Alaska Department of Fish & Game and the University of Alaska Fairbanks using the direct rapid immunohistochemical test (DRIT). However, funding for surveillance of animal rabies is currently dependent on investigator-driven research grants.

Loss of sea ice due to climate change may result in greater contact between foxes and domestic animals. It will also restrict movement of Arctic foxes. A warming Arctic is also expected to increase the range of the Red fox into Arctic fox territory, and the potential northern expansion of bats into southeastern Alaska (54). The exact consequences of these changes in the ecology of these reservoir hosts are currently not well understood (3,53,54).

## Tularemia

Tularemia results from infection with the bacterium *Francisella tularensis* through the bite of an arthropod or through an open wound while handling contaminated water or animal carcasses, from the inhalation of dust from contaminated hay or soil, or from the consumption of inadequately cooked meat of infected animals. Person-to-person transmission has not been described. After a 3–5-day incubation period (range 1–10 days), any of five different presentations of tularemia may develop depending upon the portal of entry: (1) Ulceroglandular, characterised by a painful skin lesion or ulceration with subsequent acutely enlarged and tender regional lymph nodes; the most common form. (2) Glandular with acutely enlarged and tender lymph nodes without skin lesions. (3) Oropharyngeal, severe exudative pharyngitis, sometimes accompanied by vomiting, abdominal pain or diarrhoea resulting from ingestion of the bacteria. (4) Oculoglandular severe conjunctivitis with regional lymph node involvement; the most rare form, (5) Typhoidal tularemia with pneumonia, septicaemia and hepatosplenomegaly; the most severe form. Tularemia occurs throughout North America and northern Europe, China and Japan. Reservoirs include wild or feral animals: rabbits, hares, voles, muskrats, beavers and some domestic animals. A rodent–mosquito cycle has been described (*F. tularensis* sub-species *holarctica*) in Scandinavia and Russia (55). In Alaska, both *F. tularensis* sub-species holarctica and the more virulent subspecies tularensis have been isolated from patients (56). Humans are infected via the bite of an arthropod (commonly ticks in North America and mosquitos in northern Europe) or by inoculation through the skin, conjunctiva oropharyngeal mucosa via contaminated water, blood, and tissue while handling infected carcasses. Other routes include the ingestion of contaminated meat from infected animals or by the inhalation of contaminated dust from soil, grain, hay or animal pelts.

In Alaska, human cases are reportable to the SOE and animal and wildlife cases to the OSV (57). From 1946–2009, there have been 29 human cases of tularemia reported to the SOE. Since 2001, there have been five human cases. Four of the five resided in and were infected in Alaska. The majority of cases occurred in the summer and were likely to be related to time spent outdoors and exposure to vectors. There is no surveillance in animal or insect vector reservoirs in Alaska and cases in domestic animals are likely under-reported as animals are often treated empirically. There is a need to increase surveillance of disease in wildlife to be able to monitor the impact of climate change on epizootics events of tularemia.

The most likely effect of climate change on the transmission of tularemia in Alaska will be the impact of climate on the distribution and abundance of tick vectors and their respective rodent reservoirs. In Alaska, the tree line is moving northward together with beaver, muskrat and other rodent populations, potentially exposing human residents of these regions to new diseases such as tularemia (41).

## Conclusions and recommended actions

The increasing ambient temperature in Alaska and other regions of the Arctic may influence the incidence and distribution of zoonotic and parasitic infections in humans by changing the population density and range of wild and domestic animals and insect hosts. The public health response should include enhancing the capacity to monitor those potentially climate-sensitive infections that are most likely to have a large public health impact; investigating promptly infectious disease outbreaks that may be related to climate change; and conducting research into the relationship between climate and infectious disease emergence to guide early detection and public health interventions (58). The response should also include a communication strategy to educate human and animal health care providers emphasising the need for complete surveillance reporting (59,60). The communication strategy should include education of the general public, at risk sub-populations (hunters, trappers, subsistence users wildlife biologists, livestock farmers pet owners). At particular risk are Alaska Natives who often depend on land and sea mammals to provide a healthy affordable sustainable and culturally meaningful diet. However, such a diet may place people at risk of acquiring a zoonotic disease. For example, much of the sea and land mammals that are consumed by Alaska Natives are dried or eaten raw after freezing. While this is an economical and efficient way to prepare meat preserving both nutritionally and culturally, such methods of food preparation may carry more risk for acquiring a zoonotic disease than cooking the meat.

In Alaska, for these potentially climate sensitive pathogens, the gaps in knowledge and solutions for each pathogen tend to be similar (Table I). Surveillance for infection and disease caused by many of these pathogens in both humans and animals is incomplete. It is not clear whether this is because of under reporting or under diagnosis due to a lack of adequate diagnostics, staffing issues in remote locations and logistical difficulties associated with remote specimen collection and shipping. Surveillance evaluations for zoonotic infections all of which are potentially climate sensitive infections are needed. As part of a strategy to improve reporting to SOE and OSV, education materials should be developed and distributed to medical providers and laboratories emphasising the importance of complete surveillance and reporting.

**Table I:** Common gaps in knowledge among pathogens, recommendations for actions and technology needed to address prevention and control of these zoonotic threats in the context of a changing Alaskan climate.

Agent	Gaps	Actions Needed	Technology Needed
****Brucella****	Recent baseline levels of infection and disease in humans and wildlife unknown.	Conduct seroprevalence studies in humans and wildlife. Evaluate surveillance systems in humans and wildlife.	*Brucella* species specific antibody assays. Standardised diagnostics for different animal species. Development/validation of field filter paper blood collection systems for antibody detection.
	Role of marine *Brucella* in human and wildlife disease.	Surveillance for disease in humans and wildlife.	Marine *Brucella* species specific antibody assay.
****Toxoplasma****	Recent baseline levels of infection and disease in humans and wildlife unknown. Not a notifiable disease.	Conduct seroprevalence studies in humans and wildlife. Evaluate surveillance systems in humans and wildlife.	Development/validation of field filter paper blood collection systems for antibody detection.
	Role of predator prey cycles as a mechanism of maintenance in Arctic regions.		
****Trichinella****	Recent baseline levels of infection and disease in humans and wildlife unknown.	Conduct seroprevalence studies in humans and wildlife. Evaluate surveillance systems in humans and wildlife.	Development/validation of field filter paper blood collection systems for antibody detection. Animal species specific antibody assays.
****Giardia/Cryptosporidium****	Recent baseline levels of infection and disease in humans and wildlife unknown (Underreported).	Evaluate surveillance systems in humans and wildlife.	Development/validation of serological tests.
	Significance of molecular variation of strains and relationship to disease in humans and wildlife.		
****Echinococcus****	Recent baseline levels of infection and disease in humans and wildlife unknown.	Evaluate surveillance systems in humans and wildlife.	Development/validation of *Echinococcus* species specific tests for diagnostics and seroprevalence.
	Significance of molecular variation of strains and relationship to disease in humans and wildlife.		
	Status of dog treatment programmes unknown.	Assessment of dog treatment programmes.	
***Rabies***	Prevalence in wildlife reservoirs unknown. No systematic surveillance in fox populations. Ecology and interaction between Red and Arctic fox unknown. Role of climate change on disease prevalence and host species incursion.	Evaluation of Direct Immunohistochemical Test (DRIT).	
****Tularemia****	Recent baseline levels of infection and disease in humans and wildlife unknown.	Evaluate surveillance systems in humans and wildlife.	
***All Agents***	Inconsistent disease awareness and potential role of climate change at community, tribal, health organisation levels.	Provide necessary education, outreach and communication.	
	Inconsistent communication, collaboration between subject matter experts at federal, non-federal agencies, institutes and organisations.	Convene a working group to provide communication, monitor activities, actions needed and progress.	

The challenge in Alaska will be to ensure sufficient public health capacity to allow the detection of disease outbreaks and to monitor infectious disease trends most likely influenced by climate. Much can be learned about the relationship between climate and weather and infectious disease emergence by promptly reporting food and water borne outbreaks that may be climate or weather related allowing for appropriate preventive measures to be implemented (61). However, additional resources and training may be needed to ensure adequate numbers of trained staff are available to address the emerging public and wildlife health impacts posed by climate change.

Baseline levels of infection in humans and animals are unknown. In Alaska, there is the Alaska Area Specimen Bank, which is a large collection of biologic specimens (mostly sera) collected by public health agencies since 1967. This collection can be used to determine the seroprevalence of infectious diseases of concern (62). Pilot seroprevalence studies are underway in selected communities to determine the baseline seroprevalence of *Brucella*, *Fransicella*, *Toxoplasma*, *Trichinella*, *Giardia*, *Cryptosporidium* and *Echinococcus*. Similar studies, using previously collected banked specimens need to be conducted in appropriate wildlife species.

There is a need to improve diagnostics. Current serologic tests for many pathogens of concern are not species-specific and often cross-react with other bacteria complicating their interpretation and hindering our understanding of their ecology in nature. Efforts are underway to establish and validate simple filter paper blood collection systems that can be used by hunters in the field. Such samples could be used for the detection of antibodies using serologic methods and pathogen specific DNA by molecular methods. DNA sequencing methods allows for the detection of molecular variation among pathogen strains suggesting that certain assemblages are more likely to be associated with specific animal species, i.e. parasites found in humans are more likely to align with assemblages consisting of isolates from other humans, as opposed to parasites from non-human animal species. These methods could greatly increase our understanding of disease ecology of these pathogens in humans and animals living in Alaska. More research is needed not only to understand the disease ecology of these and other emerging pathogens in humans and animals living in Alaska, but also to understand the impact of climate change on disease occurrence in humans and wildlife.

Ecosystem health, wildlife health and human health are interconnected and there is a need to develop a coordinated plan to monitor projects and activities that focus on the impact of climate change on zoonotic and parasitic infections in Alaska. This can be facilitated by forming a working group that would include representation from State of Alaska Departments of Health and Social Services, Environmental Conservation and Fish & Game; Alaska Native Tribal Health Consortium, University of Alaska, the CDC's Arctic Investigations Program, the US Fish & Wildlife Service and others to monitor activities and ensure coordination and sample sharing between appropriate agencies, institutes and organisations.
